# Improving solar PV performance under bird-dropping conditions with a dual-cooling approach

**DOI:** 10.1038/s41598-024-84932-w

**Published:** 2025-03-10

**Authors:** Khaled Abdeen Mousa Ali, Yasser Kamal Osman Taha Osman, Gomaa Galal Abd El-wahhab, Taha Abdelfattah Mohammed Abdelwahab, Ahmed Elsayed Mahmoud Fodah

**Affiliations:** 1 College of Agricultural Engineering, Al-Azhar University, Cairo, 11768 Egypt; 2 School of Energy and Power Engineering, Huazhong University of Science and Technology, Wuhan, 430074 China

**Keywords:** Thermal efficiency improvement, Solar PV panels, Dual-cooling technique, Bird dropping mitigation, IR thermal imaging., Environmental sciences, Energy science and technology, Engineering

## Abstract

The degradation performance of solar photovoltaic (SPV) panels, is a critical issue for its adoption. The current study introduces a novel dual-cooling technique to enhance the performance of the SPV panels under conditions of contamination from bird droppings. The front and backside temperatures, output power, and efficiency of the cooled SPV panels were evaluated and compared. Results showed that the cooling process reduced the front and backside temperatures by 24–47% and 34–48% respectively, compared to contaminated SPV panels. The cooled SPV module exhibited an output current increase of 8–9% and an output voltage increase of 7–9% compared to both contaminated and controlled modules. Consequently, output power for the cooled SPV module increased by 12–33% and 7–12% compared to bird droppings and controlled modules, respectively. Moreover, the overall efficiency of the SPV module dropped to 15% in the presence of bird droppings, compared to 20% with the cooling process was applied. These findings suggest significant potential benefits for large-scale SPV installations, enhancing performance and efficiency.

## Introduction

Solar energy was historically viewed as a viable alternative to a sustainable power source^1^. Recently renewable energy has gained popularity due to the growing shortage of fossil fuels in addition to its harmful effects on the environment^2,3^. This has increased the importance of and need for electric power. It is common knowledge that fossil fuels, including coal, oil, and natural gas, are finite and highly polluting energy sources that provide most of the world’s energy. Where it currently accounts for 80% of the energy supply^4^. Because of the problems above, researchers have been attempting to examine the viability of alternative power sources obtained from renewable energy resources for several decades.

With fossil fuels running out quickly, solar energy is seen as a viable renewable energy source and one of the main answers to the world’s constantly increasing energy demand. It is anticipated that in the future decades, solar energy will supplant conventional nonrenewable energy sources as the main energy source^1,5^. Solar photovoltaic (SPV) technology is emerging as the most promising harvesting method and the fastest-growing clean and renewable energy technology in Egypt by taking advantage of the country’s copious free sun radiation.

The operating temperature, the surrounding environment, the semiconductor band gap, solar radiation, and the materials used in the module are the key factors affecting a photovoltaic module’s performance. It is estimated that during operation, 17% of incident solar radiation is absorbed for power while the remaining portion is wasted as heat^6^. Additionally, the heat in the surrounding air degrades the efficiency and power output of solar panels by increasing the temperature of their cells^7^.

The weather has a significant impact on the temperature at which SPV modules operate in Egypt, especially in the summer, according to Odeh and Behnia^8^. This might cause intrinsic issues like damage or lower productivity. Therefore, increasing the efficiency of the SPV modules is one important goal that can be achieved by lowering their temperature. According to Beemkumar et al.^9^, the power yield of an SPV panel will drop from 0.2 to 0.5% for every degree it is heated, indicating the urgent necessity for a cooling procedure.

As a result, maintaining the SPV modules’ functionality depends on cooling them. Cooling the SPV panel would provide a good solution to this problem if it could be implemented efficiently. Air, water, refrigerant, and phase-change materials are the most common cooling mediums used so far^10–16^. Different cooling techniques are applied in the literature to reduce the temperatures of the SPV panels and increase their output power^17^. Cooling techniques can be applied to the top surface, bottom surface, or both surfaces of the SPV^18^. Tuncer et al.^19^ used paraffin wax-filled aluminum cans for thermal management in photovoltaic systems, improving the electrical efficiency of PV panels from 10.69 to 12.60% and increasing normalized power output efficiency from 61.72 to 71.56%. In their comprehensive study, Abo et al.^20^ found that water-cooling techniques significantly enhanced photovoltaic panel efficiency, with improvements ranging from 6 to 82.6%, depending on the method. A water spray cooling system, particularly effective in dusty environments, helped maintain low surface temperatures and kept the panels clean, optimizing performance. Additionally, evaporative cooling boosted efficiency by 7.6–23% without requiring any pumping power. Yousef et al.^21^ found that using a front surface water cooling system reduced the temperature of SPV modules by 25%, leading to a 14% increase in output power. The same results also have been reported by Moharram et al.^22^. Authors of^23^ observed a 12.5% increase in SPV module efficiency with front surface water cooling compared to the control (no cooling). Bilen and Erdoğan^24^ also studied the effect of front surface water cooling on SPV modules, finding a 16.3% increase in power output and a reduction in module temperature to 24 °C. Authors of^25^ innovated a mathematical model that examines the thermal properties of heat sink-assisted elevated temperature sintering for TiO_2_-coated polymer photogenic trade, demonstrating its potential to enhance the performance of flexible dye-sensitized solar cells (FDSSCs) by effectively sintering TiO_2_ films on polymer-surfaces.

Alami^26^ investigated the effect of evaporative cooling on photovoltaic module efficiency by covering the bottom of the SPV module with a clay layer, creating a gap. Water passed through the gap, lowering the module’s temperature. This cooling system increased the module’s maximum output power by 19%. Hasan et al.^27^. examined evaporative cooling using pin fans and a moist wood wool pad, which reduced the module’s temperature from 61 °C to 45 °C. This led to a 32.7% increase in output power and a 31.7% improvement in efficiency. Haidara et al.^28^ used a wet cloth on the backside of the SPV module, reducing the temperature by more than 20 °C and increasing output power by 14%.

The primary causes of soiling on SPV systems are various particulate matters from construction locations, farming operations, air pollution, and bird droppings^29^. Due to their shading effects, bird droppings on the SPV module surface reduce their output power production. However, removing bird droppings from the surface is difficult when the SPV systems are installed in remote or offshore areas^29,30^. Bird droppings also contain digestive fluids that heavy rains can’t cleanse. Therefore, the quality of covering the surfaces of SPV modules with bird droppings must be known to prepare them for design when installing photovoltaic systems in polar regions, remote areas, and marine environments^29^. Bird guano accumulated on the SPV modules decreasing the performance of solar panels by reducing the transmittance of the glass cover on the PV panels. Sisodia and Mathur^31^, studied the impact of bird droppings on the performance of SPV modules. They found that bird droppings significantly reduce output power by 23.8%. The study evaluated the thermal behavior of modules with varying amounts of droppings (1–4 drops) compared to a clean module. Results showed that bird droppings create hot spots, increasing module temperature by 5%. A higher accumulation (4 drops) decreased module current by 36–38%, while voltage remained minimally affected^32^.

## **Materials and methods**

Hadipour et al.^33^ The levelized cost of electricity by the uncooled system was found lower than the spray-cooled systems but very near to pulsed-spray water cooling with DC = 0.2. The levelized cost of electricity produced by the PV system is reduced by about 46.5% and 76.3% by using the pulsed-spray water cooling system with DC = 1 and 0.2, respectively as compared with the case of steady-spray water cooling system. As a result, the new pulsed-spray water cooling is efficient from the economic point of view. As previously mentioned, several research have been conducted to determine how top and bottom surface cooling systems affect the SPV modules’ efficiency^26–28^. Furthermore, while previous studies have explored various cooling methods for SPV panels, including air, water, and evaporative cooling, most of these investigations have been conducted in laboratory settings or without considering the effect of bird droppings on panel performance. Furthermore, there is a lack of studies examining dual-cooling techniques, especially in real-world outdoor conditions where panels are exposed to environmental factors like dust and bird droppings. This research aims to address these gaps by developing and testing a novel dual-cooling method that integrates both front and back cooling systems while also cleaning the front surface. The study utilizes infrared thermography to provide precise measurements of temperature and performance, offering a more accurate assessment of SPV module efficiency in real outdoor environments.

### Cooling system setup

**Fig. 1:**
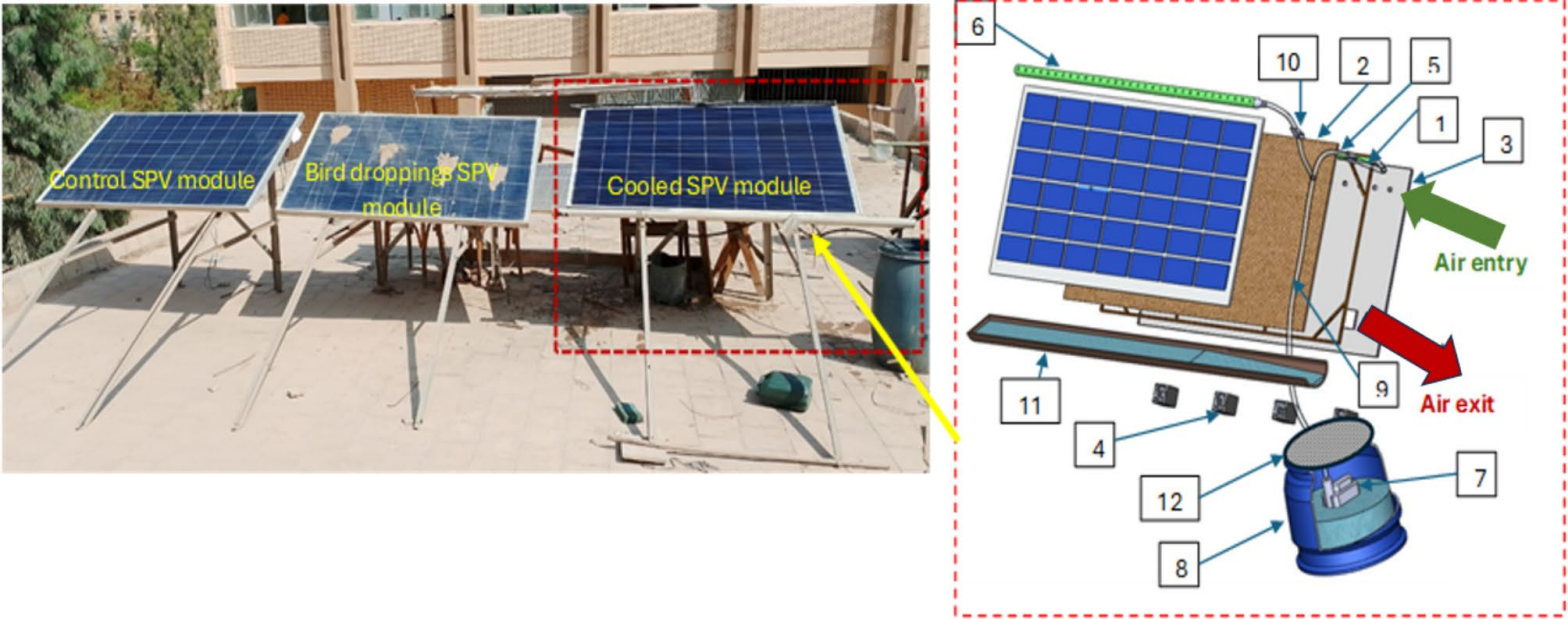
Experimental setup for (left) Complete setup; (right) Schematic diagram for front and backside cooling: 1- wooden frame 2- cotton fabric 3- Acrylic sheet 4- Four axial fans (Air exit) 5- Inner water pipe 6- Outer pipe 7- submersible pump 8- Reservoir 9- PVC pipes 10- Valve 11- Water collector 12- Filter.

Applying the dual-cooling system for SPV modules can promise a significant potential for improving the performance and longevity of solar energy systems, particularly in regions with high temperatures or when the modules are exposed to contamination such as bird droppings. A novel cooling system has been fitted together and operated, as Fig. 1 illustrates. The three components of the cooling system are a water collector placed on the bottom side of the SPV module. The specifications of the tested solar SPV modules are listed in Table 1. A cleaning unit is placed on the front side, and an evaporative cooling unit is placed on the rear side of the SPV module. On August 10, 2023, a clear and sunny day, the experiment was run nonstop for eight hours.

**Table 1:** The used SPV modules’ specifications.

Solar module type	Multi-crystalline silicon
Peak powers	360 W
Max. power voltage	37.6 V
Max. power current	9.58 A
Open-circuit voltage	45.8 V
Short-circuit current	10.18 A
Dimensions	1755 × 1038 × 33 mm

As illustrated in Fig. 1, the evaporative cooling unit is composed of a wooden frame, cotton fabric, an acrylic sheet, four axial fans, and an inner water pipe. A wooden frame measuring 1755 × 1038 × 33 mm is fastened to the rear of the SPV module. A 1 mm thick piece of cotton fabric with dimensions of 1755 × 1038 mm was fixed to the side of the wooden frame opposite the back of the SPV. A 6 mm thick acrylic sheet was fixed to the other side of the wooden frame. The distance between the acrylic sheet and the cotton fabric is ~ 1 cm to allow air exchange by using four axial fans with a diameter of 8 cm (12 V, 1.8 W, air flow of 43 CFM, and speed of 2800 rpm) through 15 holes with a diameter of 1.2 cm at distances of 10 that were made on the upper side of the acrylic sheet. On the upper section of the wooden frame, a central water pipe with a diameter of 2.54 cm was installed, featuring closely spaced perforations of 2 mm in diameter. Water spreads when it falls on the fabric through capillary action. Air is drawn in through fans, where the air is saturated with moisture, so the temperature of the air between the fabric and the surface of the solar panel decreases. Heat is transferred from the panel to the air, and the temperature of the solar panel decreases.

The cleaning unit has two functions: cooling and cleaning. It is composed of an exterior pipe (1.6 cm in diameter) positioned horizontally on top of the SPV module border. To guarantee complete water coverage throughout the SPV module, holes are spaced 2 cm apart. With the help of PVC pipes that have two valves to control water flow, the outer and inner pipes are connected to a submersible pump (12 V, 19 W, maximum flow rate 500 L/h, and maximum water head of 9 m) that is located inside a 50 L reservoir. At the start of the experiment, water was added to the tank. To make up for the water deficit, water is periodically added to the tank.

A water collector was installed on the bottom side of the SPV module to collect the water from the cleaning and the evaporative cooling units.

### Instruments used

Backside, and frontside temperatures (℃), module output current (A), module voltage (V), sunlight intensity, ambient temperature (℃), and relative humidity (%) were measured for the three SPV modules from 10 AM to 5 PM. The specifications and technical details of the used devices are listed in Table 2.

To assess the thermal behavior of the SPV module’s back surface, five K-type thermocouples mounted to each module’s back surface and linked to an Arduino Mega 2560 Rev3 board were used. The Raspberry Pi board (4 B-8GB), which stores all temperature readings and quickly processes data thereafter, was attached to the Arduino board. For monitoring the thermal behavior of the SPV module’s front surface, an infrared thermography (IRT) camera was employed. This approach was necessary due to the challenges posed by the reflection of chassis temperature in thermography, which can interfere with accurate image processing.

**Table 2:** Technical specifications of the used devices.

Thermocouple	Type	K-Type
	Range	0–125 ℃
Infrared thermal imager	Model	Uti 260B
	Temperature range	-15 ~ 550 ℃
	IR resolution	× 192 pixels
Solar power meter	Type	TM-207
	Resolution	1 W/m^2^
	Accuracy	± 10 W/m^2^
Digital environmental meter	Model	UT333
	Relative humidity range	0-100%
	Accuracy	± 0.1%
	Temperature range	0–60 ℃
	Accuracy	± 1%
Multimeter	Type	BM822A
	DC Voltage range	400 Mv-600 V
	Accuracy	± 1%
	DC Current range	400–600 A
	Accuracy	± 2%

### Photovoltaic characteristics and solar testing

The buildup of bird droppings on photovoltaic solar modules creates “hot spots,” on the panel surfaces, raising their temperature by 5% compared to clean modules. This temperature increase reduces current output by 36–38% when there are four droppings or more^32^. Three SPV module types, multi-crystalline silicon 360 W, were used with dimensions of 1755 × 1038 × 33 mm on a flat roof as shown in Fig. 1. The first SPV module was equipped with an innovative cooling system (named the cooled SPV module); the second SPV module was subjected to four bird droppings (named the bird droppings SPV module)^32^; while the third SPV module was completely clean for comparison (named as cleaned or controlled SPV module).

The output power of the SPV modules ($\:{\text{P}}_{\text{P}\text{V}}$) has been calculated depending on the average values of the current ($\:{\text{I}}_{\text{P}\text{V}}$) and voltage ($\:{\text{V}}_{PV}$) from Eq. (1). Thus, to estimate the module efficiency, the SPV module area ($\:\text{A}$) and the average values of the solar radiation ($\:\text{S}$) are considered, as seen in Eq. (2).

1 $$\:{\text{P}}_{\text{P}\text{V}}={\text{V}}_{PV}\times\:\:{\text{I}}_{\text{P}\text{V}}$$

2 $$\:{\upmu\:}=\frac{{P}_{PV}}{\text{A}\times\:\text{S}}$$

Fill factor (FF) is the parameter that defines the quality of the SPV module, which represents the maximum power conversion efficiency of the SPV module that can be calculated from **Eq. (3)**^34^.

3 $$\:\text{F}\text{F}=\frac{{V}_{PV}\:\times\:\:{I}_{PV}}{{V}_{OC}\:\times\:\:{I}_{SC}}$$

Where: $\:{V}_{OC}$ is open circuit voltage (V); $\:{I}_{SC}$ is short circuit current (A).

**Fig. 2:**
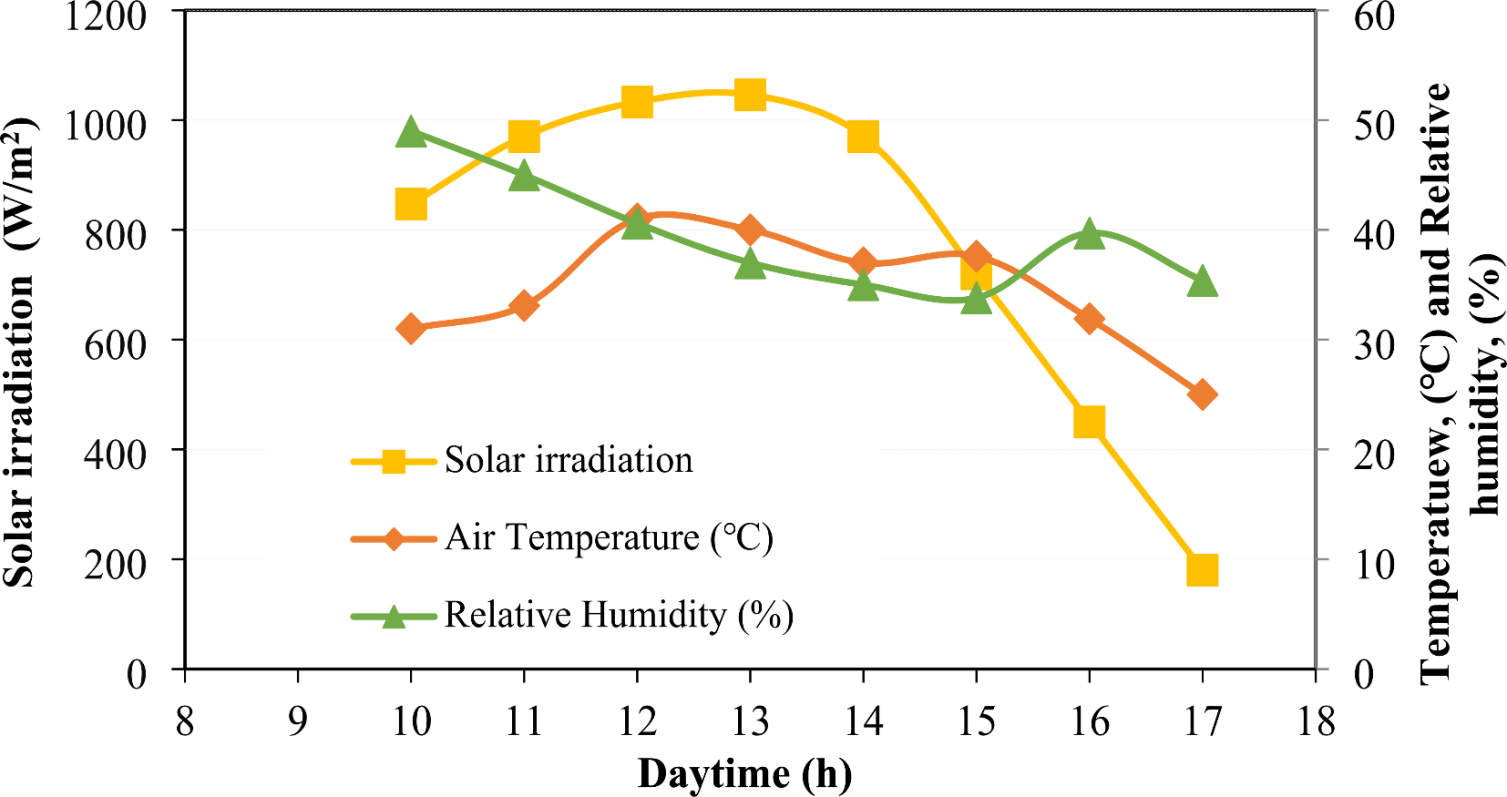
Outdoor conditions during the experiments.

The experiments were carried out taking into account Cairo, Egypt’s climate. The climate of Egypt is desert-like. The majority of the year is hot and dry. Egypt’s capital city, Cairo, can be found at (30.04167° N, 31.23528° E). Cairo experiences 4.7 kWh/m^2^/day of solar radiation and 22.1 °C on average annual temperature. The yearly wind speed is 4.02 m/s, while the average annual humidity is 55%. Figure 2 depicts the specific outdoor conditions on August 10, 2023, during the day of the testing. August is known for its bright days, high temperatures, mostly low humidity, and mild winds. The modules had a 30° lean at Cairo latitude and were oriented southward.

### Data calculation

The Data Reduction Uncertainty Analysis was conducted to assess the reliability of experimental measurements. Its main goal is to verify the accuracy of the recorded data during experiments and to estimate the uncertainties involved in measuring the output power and efficiency of the SPV (Solar Photovoltaic) module. Specifically, the uncertainties considered in this analysis arise from the measurements of voltage, current, and solar radiation. The uncertainty analysis was carried out using the Coleman and Steele method^35^. The method is applied to estimate the uncertainties in output power and the efficiency of the SPV modules.

Uncertainty in Output Power: The uncertainty in output power (P_out_) is primarily influenced by uncertainties in two key variables: voltage (V) and current (I). These were the main measurements used to calculate the output power of the SPV module. The uncertainty in P_out_ is then calculated by propagating the uncertainties in voltage and current the seen in Eq. (4). Which estimates the total uncertainty by considering how errors in V_PV_ and I_PV_ combine to affect P_out_. The final uncertainty in output power was found to be ± 0.062%.

4 $$\:\frac{{\varDelta\:P}_{PV}}{{P}_{PV}}={\left[{\left(\frac{{\varDelta\:V}_{PV}}{{V}_{PV}}\right)}^{2}+{\left(\frac{{\varDelta\:I}_{PV}}{{I}_{PV}}\right)}^{2}\right]}^{0.5}$$

Uncertainty in Efficiency: The uncertainties in efficiency arise from the uncertainties in output power (Pout), solar radiation (S), and area (A). To calculate the overall uncertainty in efficiency, we used the propagation of uncertainty formula, which combines the uncertainties of these three variables. This can be expressed as seen in Eq. (5). After applying this method, the uncertainty in efficiency was found to be ± 0.32%.

5 $$\:\frac{\varDelta\:\mu\:}{\mu\:}={\left[{\left(\frac{{\varDelta\:P}_{PV}}{{P}_{PV}}\right)}^{2}+{\left(\frac{\varDelta\:A}{A}\right)}^{2}+\:{\left(\frac{\varDelta\:S}{S}\right)}^{2}\:\right]}^{0.5}$$

Where: $\:\varDelta\:\mu\:$ is the uncertainty in efficiency, $\:{\varDelta\:P}_{PV}$ is the uncertainty in output power, $\:\varDelta\:A$ is the uncertainty in area, and ΔS is the uncertainty in solar radiation.

### Cost’s calculations

The following equation was used to convert the cost per square meter to cost peak watt^36^:

6 $$\:\frac{\$}{{W}_{p}}=\frac{\$/{m}^{2}}{\eta\:1000{W}_{p}/{m}^{2}}$$

In this study, the peak solar irradiance is 1000 W/m^2^, and a photovoltaic panel with a cost of 120 $/m^2^ is used. Accordingly, the cost per peak watt is 1 $/Wp for the investigated mode with an efficiency of η = 12%.

As a fundamental economic concept for each PV system, the costs should be recovered by the useful energy produced by the system over its lifetime. The levelized cost of electricity (LCOE) is defined as the ratio of the total life cycle cost to the total lifetime energy production, based on the following equations^36–38^.

7 $$\:LCOE=\frac{\left(Anuual\:cost+\:O\&M\right)\$}{Anuual\:output\:cost\left(kWh\right)}$$

The Levelized Cost of Electricity (LCOE) is a key economic concept used to evaluate the cost-effectiveness of energy production from a system, such as a photovoltaic (PV) system. It provides a way to compare the cost of electricity from different energy sources, regardless of their characteristics. The LCOE gives a measure of the cost per unit of energy (typically in cents per kWh) that a system generates over its lifetime. The LCOE is defined as the ratio of the total lifetime cost of the PV system to the total lifetime energy production (i.e., the total amount of electricity generated by the system over its entire operational life). This helps determine the cost-effectiveness of the system and whether the energy produced justifies the investment.

Annual output = Average Annual Insolation × Efficiency

$$Average\; Annual\;Insolation = \frac{{5.5KWh}}{{day m^{2} }} \times \frac{{365day}}{{year}} = 2007KWh$$

Annual Cost = (Installation Cost × CRF) + water cost + (cooling system cost × CRF) + O&M (O&M = 3% of installation cost per year).

Installation Cost = Capital Cost × Station Capacity = $120.

Station Capacity = 1 m^2^.

Capital Cost = $160/m^2^ or ($1.3/W).

The following equation is used to calculate the capital recovery factor (CRF) for the PV systems^39^:

8 $$\:CRF=\frac{i{(i+1)}^{n}}{{(i+1)}^{n}-1}$$

Where: n is the life of the panel, i is an interest rate.

The equation provided is used to calculate the Capital Recovery Factor (CRF) for photovoltaic (PV) systems. The CRF is a financial factor that helps determine the annual cost required to recover the capital investment made in the system over its expected lifespan. It is an important part of cost-benefit analysis for PV systems, as it helps understand how much money needs to be recovered each year to justify the investment.

## Results and discussions

This investigation aims to assess the impact of a new cooling technique on both sides of the solar photovoltaic (SPV) modules. The performance of SPV modules specifically front and backside temperatures, output power, and module efficiency has been detected, recorded, and compared for bird droppings, controlled, and cooled SPV modules under cloudless outdoor conditions. This study focused on three SPV modules exposed to outdoor conditions in Cairo, Egypt. The details of outdoor conditions during the experiments are shown in Fig. 2.

### Front and backside temperature of SPV modules under bird droppings and cooled conditions

**Fig. 3:**
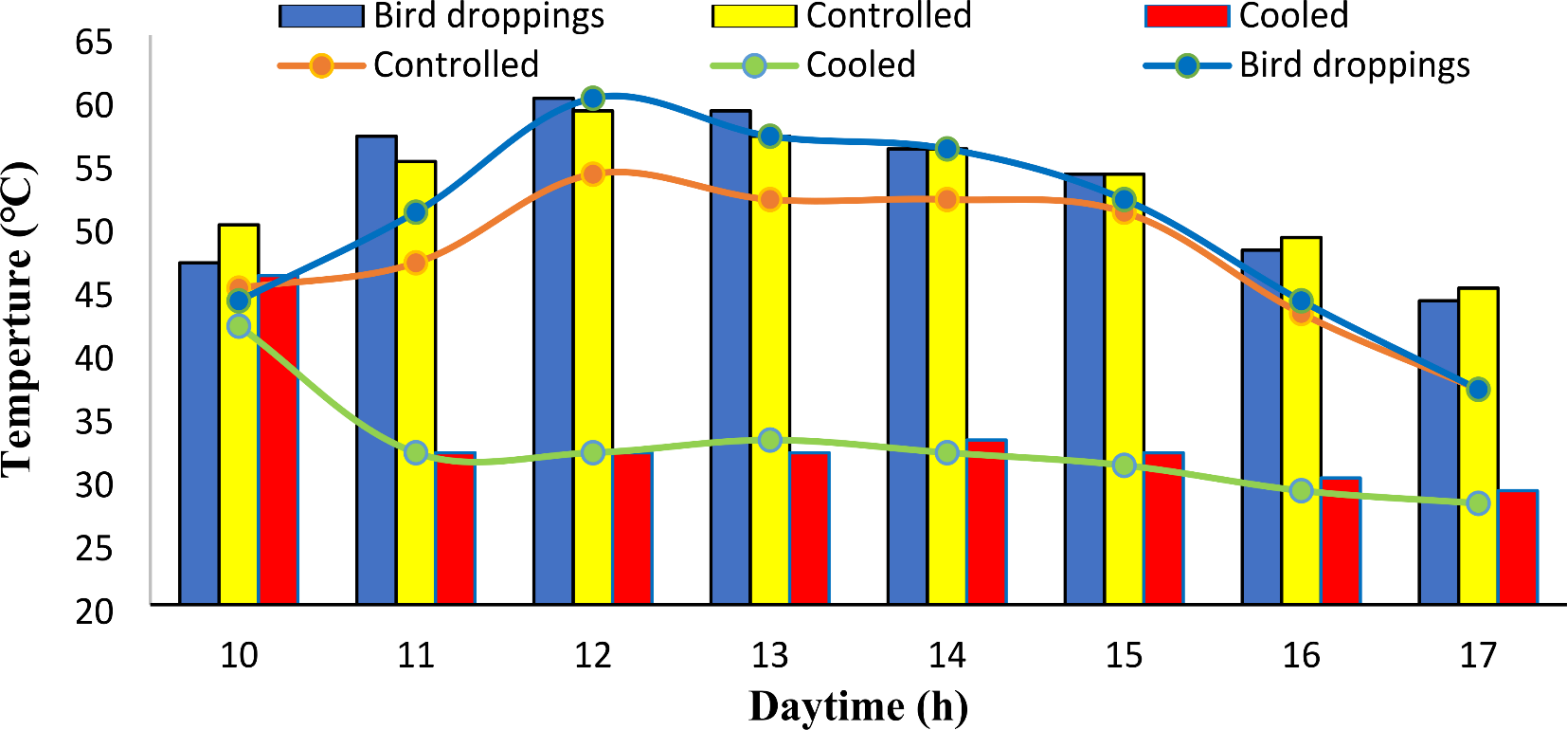
The daytime frontside (line) and backside (column) temperatures for different solar photovoltaic modules under different conditions.

Figure 3 shows the relation between daytime frontside (line) and backside (column) temperatures for different solar photovoltaic modules under bird droppings and cooled conditions. The front temperature of the solar panels was measured with the help of an IRT camera (Sect. 2.2).

From Fig. 3 it was noticeable that, for the frontside and backside temperatures of the SPV modules, the working temperatures were found to increase markedly with the daytime from 10 am to a peak at 12–13 noon then decrease. This is normal because the maximum solar radiation occurrence at 12–13 noon, as reported in Fig. 2, caused more generation, which led to an increase in the temperature of the SPV modules^27^. Also, the frontside temperatures of bird droppings and cooled SPV modules were approximately equal at the beginning of the experiment (at 10 am). The same was also recorded for their backside temperatures. This is because the cooled SPV panel was covered with the same bird droppings accumulated before the beginning of the cooling process (both conditions are the same).

From Fig. 3, regarding the frontside temperature, the frontside temperature of the cooled panel was significantly lower compared to bird droppings and control SPV modules. The cooled panel exhibited a maximum recorded temperature of 42 ℃ at 10 am. This is normal because the cooling process has not begun yet at 10 am. When the cooling process was initiated, the frontside temperature of the cooled panel gradually decreased with running daytime and recorded its minimum values of 29 and 28 ℃, at 4 pm and 5 pm respectively. This phenomenon was because of the increase in solar radiation, which reached a maximum at noon and then decreased at 5 pm (Fig. 3). In contrast, the frontside temperatures of the SPV panel with bird droppings and controlled SPV panel were higher by 24–47% and 23–40%, respectively, as compared with the cooled SPV panel.

**Fig. 4:**
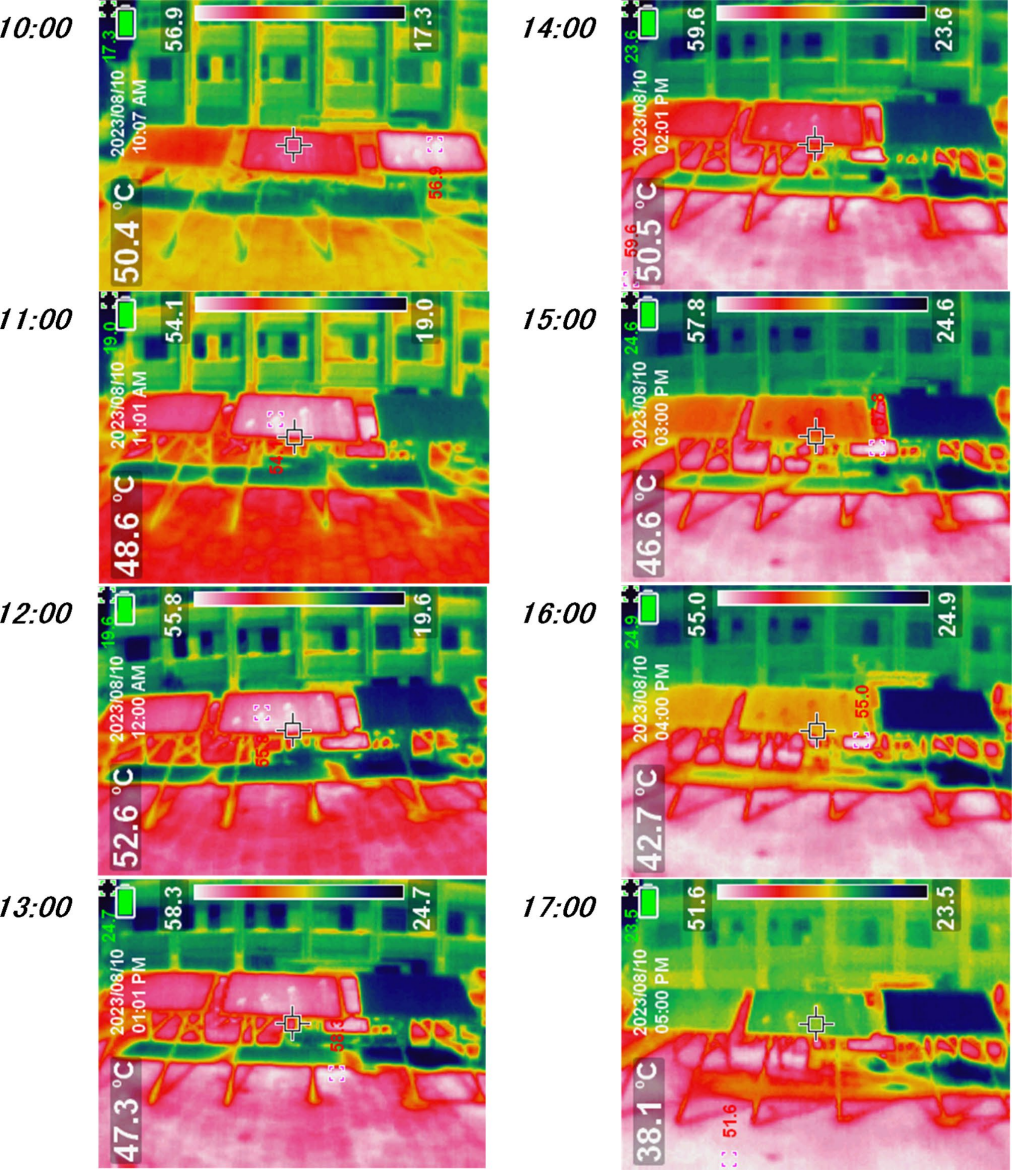
The thermal images for the cooled (Right), bird droppings (Middle), and controlled (Left) SPV during day hours.

For the backside temperature of the SPV modules, as shown in Fig. 3, the temperature of the cooled SPV module was found to be lower compared to the corresponding temperatures observed in bird droppings and controlled modules. At 10 am, the backside temperature of the modules recorded temperatures of 46 °C, 50 °C, and 47 °C for the cooled, controlled, and bird droppings SPV modules, respectively. Then, the temperature of the cooled module declined reaching an average range of 29–33 °C in the daytime due to the continuous cooling process. Conversely, the temperatures recorded on the bird droppings and controlled SPV modules continued to rise, reaching their maximum values at 12–13 noon, which were found to be 59–60 °C and 57–59 °C for bird droppings and controlled SPV modules, respectively. Also, the minimum values of the backside temperature of the three SPV modules were recorded at 5 pm due to sunset time. In contrast, the backside temperatures of the bird droppings and controlled SPV panels were higher by 34–48% and 35–45%, respectively, as compared with the cooled SPV module. Moreover, the backside temperature of the bird droppings module was the highest due to the presence of the bird droppings on the panels especially at noon time^28^. This phenomenon may be because, the bird droppings cause trapping and concentrate of the heat inside the SPV modules casing the hotspot on the module^31^, as seen in Fig. 4.

It is notable that, from Figs. 3 and 4, the frontside and backside temperatures for the cooled SPV module were found to be approximately equal over the daytime. This is because of the effective applied cooling process for both sides.

### Current-voltage curves of SPV modules under bird droppings and cooled conditions

**Fig. 5:**
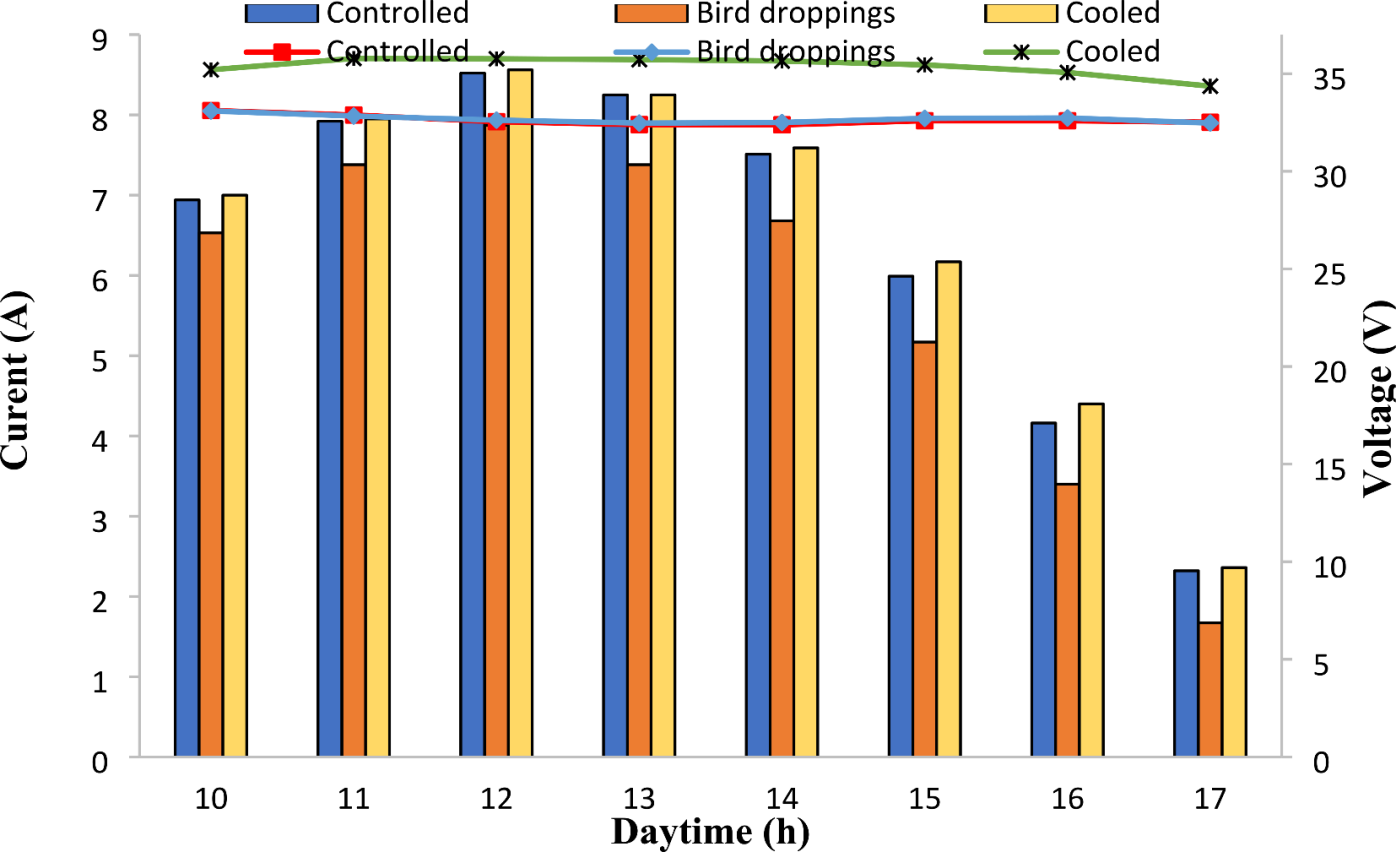
Current (lines) and voltage (columns) characteristics of solar photovoltaic modules under bird droppings and cooled conditions.

Figure 5 describes the current-voltage characteristics of SPV modules under bird droppings and cooled conditions during the daytime. Figure 5 elucidates important information for a better understanding of the degradation that occurs in the current-voltage characteristics of SPV modules under bird droppings and cooled conditions.

As can be seen from Fig. 5, for all SPV modules, the output currents were found to be increased slightly during the daytime from 10 am to 12 noon then decreased sharply with the time up reaching a minimum value at sunset (5 pm). This is normal because the maximum solar radiation occurs at noon (Fig. 2). On the other hand, the voltage of the SPV modules was found to be slightly affected by the solar radiation during the daytime from 10 am to 5 pm except for the SPV module with bird droppings which was found to be affected by the sunset.

As can be seen from Fig. 5, the output current of the cooled SPV module was found to have the highest value compared to the corresponding reading for the bird droppings and controlled SPV modules. The remarkable reduction in output current from bird droppings SPV module compared to other SPV modules (cooled and controlled) represents the effect of bird droppings deposition on the SPV modules’ surfaces. The bird droppings accumulated on the SPV led to an increase in its backside temperature (Figs. 3 and 4) resulting in a reduction in its output current^8^. It is noteworthy that the output current of the cooled SPV module was found to be higher by 1–2% and 8–9% compared with the controlled and bird droppings modules, respectively.

On the other hand, the output voltage of the cooled SPV module was found to be higher by 7–9% compared to the controlled and bird droppings modules, which is distinctively attributed to the impact of the cooling process. Furthermore, there was no remarkable difference between the output voltage from controlled and bird droppings SPV modules. It is noteworthy that the enhancement of the output voltage from the cooled module is caused by the reduction in its backside temperature by the continuous cooling process^32,34^. It is important to note that the cooled SPV module displayed a distinct voltage profile, recording a value of 35.8 V at noon and compared to corresponding values of 32.5 V for bird droppings and controlled modules. This distinctive cooling impact facilitated an enhancement in the module output voltage, especially at noon.

### Output power and efficiency of SPV modules under bird droppings and cooled conditions

**Fig. 6:**
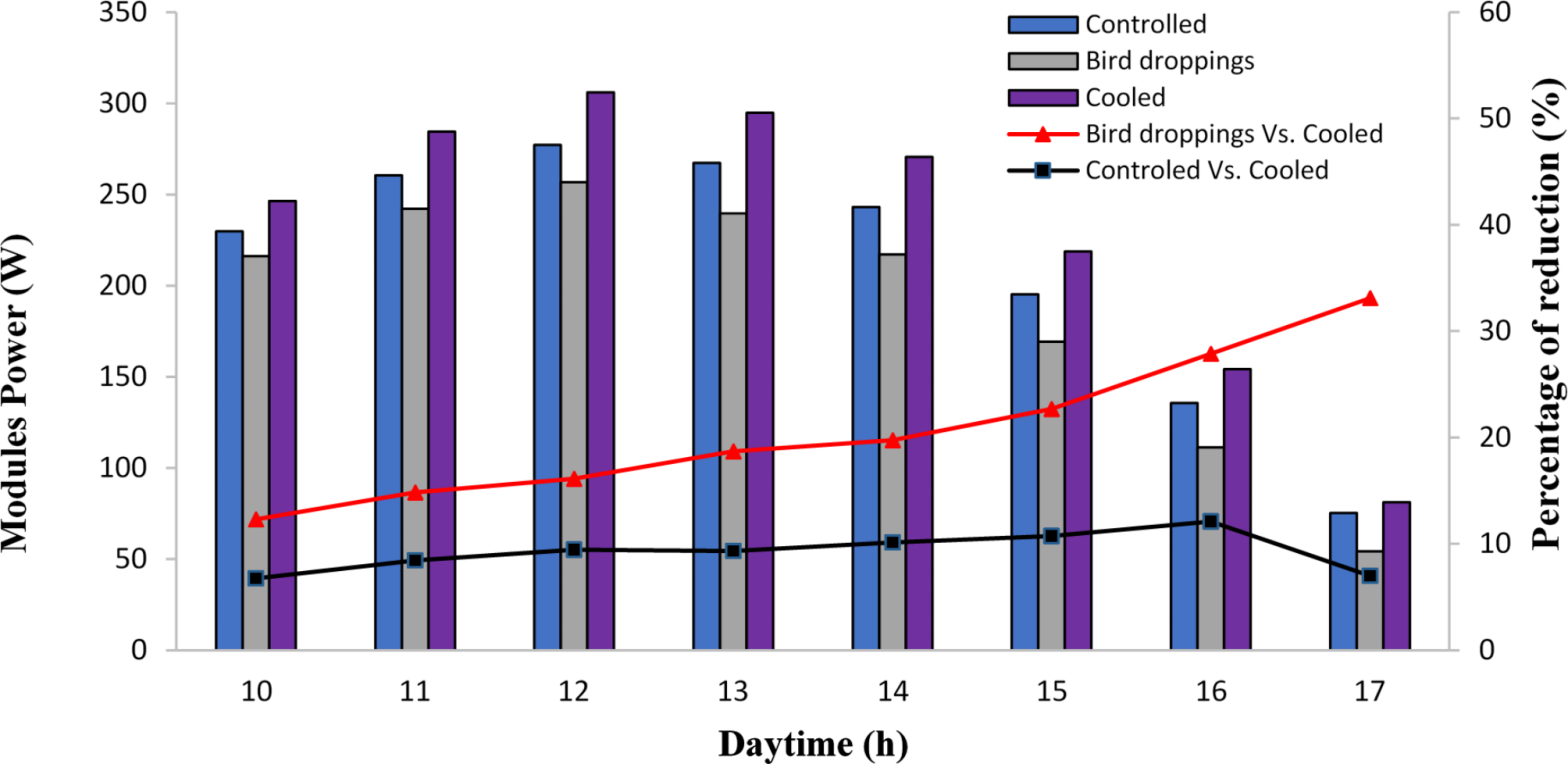
Effect of cooling process on output power of solar photovoltaic modules: (output power; column; and percentage of reduction as compared with clean module: Lines).

Figure 6 illustrates the output power of cooled, bird droppings, and controlled SPV modules during the daytime. As can be seen from Fig. 6, the output power of the SPV modules exhibited a remarkable increase from 10 am up to reaching its peak at 12 noon, then decreased sharply with time reaching the minimum values at 5 pm. This behavior is expected because the highest solar radiation is received on the SPV modules at noon (Fig. 2), causing an increase in generating output current^40^. Furthermore, from Fig. 6, the output power increased by 24%, 18%, and 21% for cooled, bird droppings, and controlled SPV modules, respectively, during running the daytime from 10 am to 12 noon, then decreased by 73%, 80%, and 73% at 5 pm compared to their values at 12 noon. Additionally, when comparing the SPV modules, the output power for the cooled SPV module was found to be higher by 12–33% and 7–12% compared to bird droppings and controlled modules throughout the daytime which represents the importance of the current cooling process.

This means the presence of bird droppings on the SPV modules has a remarkable effect on the reduction of the module’s power^28,32^. These results prove that the current cooling process has a remarkable effect on the output power of the SPV module compared to bird droppings and controlled modules.

**Fig. 7:**
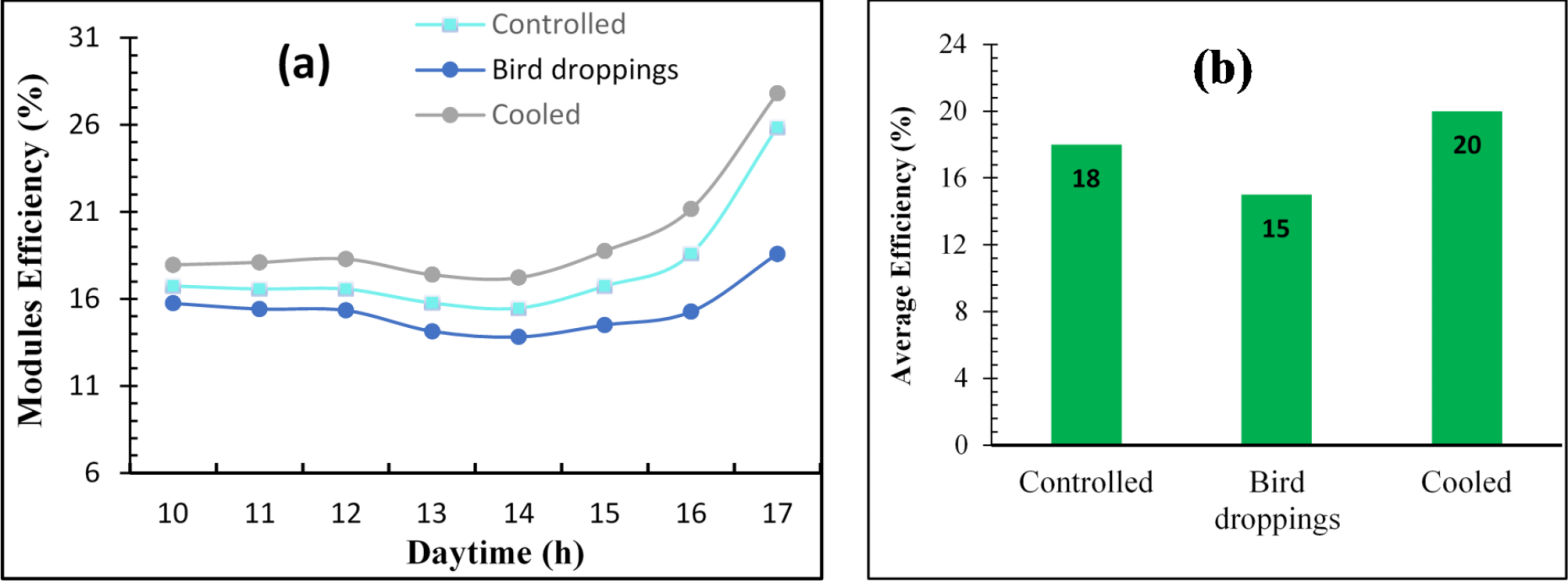
The effect of cooling process on the efficiency of solar photovoltaic modules; (a) Hourly efficiency and (b) Average efficiency.

On the other hand, Fig. 7 illustrates the impact of the cooling process on the output efficiency of the SPV module. As depicted in Fig. 7a, the hourly efficiency of the bird droppings and controlled SPV modules was observed to be lower compared to the corresponding values of the cooled SPV module. Furthermore, the presence of bird droppings on the SPV has a remarkable effect on reducing the hourly efficiency of the SPV module. It is worth noting that the SPV module with bird droppings significantly reduces the overall efficiency, up to 15% (Fig. 7b) compared to 20% and 18% for cooled and controlled modules, respectively. This disparity can be attributed to the fact that the current cooling process enhances the output power of the SPV module, resulting in higher efficiency compared to bird droppings and controlled SPV modules. As a result of the current study, the cleaning system shall be operated if the number of birds dropping exceeds 4 drops. That’s because the power reduction was high in the case of the presence of 4 no. of bird dropping on the PV panels (33% lower than cooled PV panel) which reduced the module efficiency markedly. The cooling system should also operate based on the temperature of the PV modules during the day. In the operation, we adjust the system to operate when the temperature exceeds 35 ℃ during daytime.

### Economic evaluation

A cost analysis was conducted for the proposed photovoltaic (PV) system. This analysis is important as it can determine the levelized cost of electricity (LCOE) generated by the solar photovoltaic (SPV) system^33,39,41^. In the current study, the capital cost includes all costs of the SPV system, such as the costs of farms, cables, fans, electrical cables, water pump, etc. have been assumed and involved. The operation cost of the cooling system like pumping water and operating the fans has been calculated based on the daily operation, then it has been subtracted from the output power of the cooling PV modules.

The results of this analysis for three cases are presented in Table 2. The life of the PV panel is assumed to be ten years, i.e., *n* = 10. The LCOE values produced by three SPV systems are compared in Table 3. It can be observed that by employing the proposed cooling system, the LCOE of the SPV modules is reduced by 38.46% and 8% compared to bird droppings accumulation and uncooled SPV modules, respectively. As a result, the current proposed dual-cooling system is efficient from an economic perspective. It should be highlighted that using a cooling system can eliminate hot spots on the PV panel surface and, accordingly, increase the panel’s lifetime. This is also beneficial from an economic point of view, as it can enhance the long-term viability and profitability of the SPV system. The key economic parameters considered in this analysis include the capital recovery factor (CRF), annual operating and maintenance (O&M) costs, and the annual energy output of the SPV system. The CRF accounts for the amortization of the initial capital investment over the project lifetime, while the O&M costs represent the ongoing expenses required to maintain the system’s operation. The system’s efficiency determines the annual energy output, which can be improved by implementing the proposed cooling strategy. Overall, the cost analysis demonstrates the economic advantages of the dual-cooling system in enhancing the LCOE and potentially extending the lifespan of the SPV modules, leading to improved financial performance of the PV system. As a result, the current cooling system was found to be effective in enhancing the output power and efficiency of PV modules. However, future work regarding the scaling up of the current cooling system and its effect at large-scale operation is highly recommended.

**Table 3:** The results of the cost analysis for four cases and *n* = 10.

Type of PV system	(Interest rate (%))	CRF	Capital cost ($)^a^	O&M ($)	Water cost ($)^b^	Cooling system cost ($)^c^	Annual output (kWh) ($)	LCOE ($/kWh) ($)
Uncooling system PV panel	27(10)	0.3	120	3.6	0	0	361.26	0.11
bird droppings PV panel	27(10)	0.3	120	3.6	0	0	301.05	0.13
cooling system PV panel	27(10)	0.3	120	3.6	0.18	13	401.4	0.11

In this study, all costs of the system are calculated based on the price in Egypt.

^a^The capital cost includes all costs of the SPV system, such as the costs of farms, cables, etc.).

^b^The cost of water is calculated based on the price of water in Egypt (0.02 $/m^3^).

^c^The cooling system cost includes the cost of the valve, submersible pump, reservoir, etc.

### Current research contribution, and future research directions

The application of the dual-cooling system for SPV modules, as demonstrated in this study, holds significant potential for improving the performance and longevity of solar energy systems, particularly in regions with high temperatures or when the modules are exposed to contamination such as bird droppings. The cooling system’s ability to enhance both the efficiency and output power of SPV modules can lead to substantial economic benefits, making it a valuable strategy for optimizing solar energy generation. The contribution of this study lies in offering a cost-effective solution to mitigate the detrimental effects of elevated temperatures on SPV modules, improving their performance and potentially reducing the levelized cost of energy (LCOE). However, future research should focus on assessing the long-term environmental impact, scalability of the cooling system, and its integration with other emerging technologies in solar power generation, such as energy storage and smart grid systems, to further optimize performance in diverse settings.

## Conclusion

The accumulated bird droppings and elevated temperatures from higher solar irradiance, especially at noon, have a major effect on the performance of solar photovoltaic (SPV) panels. So, this study represents a new dual-cooling technique for improving the performance of the SPV module under bird droppings and elevated temperature conditions. The performance of the cooled SPV panel was evaluated and compared. The results revealed that the cooled panel exhibited significantly lower front and backside temperatures (by 48%) compared to the panels with bird droppings. The output current of the cooled SPV module was significantly enhanced by 9% compared to with bird droppings module. The cooled panel displayed a distinct voltage profile and showcased a higher output voltage than other SPV modules. The implementation of the current cooling process caused an enhancement in the output power and efficiency of the module compared to bird droppings and controlled modules. These findings not only underscore the effectiveness of the dual-cooling technique in improving output power and efficiency but also suggest significant implications for real-world applications. By addressing common performance detractors such as bird droppings and elevated temperatures, this technique could enhance the reliability and longevity of SPV systems.

## Data Availability

Data Availability Statement: The datasets used and/or analyzed during the current study are available from the corresponding author upon reasonable request.
